# Publisher Correction: Ultrahigh high-strain-rate superplasticity in a nanostructured high-entropy alloy

**DOI:** 10.1038/s41467-022-28422-5

**Published:** 2022-02-02

**Authors:** Nhung Thi-Cam Nguyen, Peyman Asghari-Rad, Praveen Sathiyamoorthi, Alireza Zargaran, Chong Soo Lee, Hyoung Seop Kim

**Affiliations:** 1 Department of Materials Science and Engineering, Pohang University of Science and Technology (POSTECH), Pohang, 37673 South Korea; 2 Center for High Entropy Alloys, Pohang University of Science and Technology (POSTECH), Pohang, 37673 South Korea; 3 Graduate Institute of Ferrous Technology, Pohang University of Science and Technology (POSTECH), Pohang, 37673 South Korea

**Keywords:** Mechanical properties, Metals and alloys

Correction to: *Nature Communications* 10.1038/s41467-020-16601-1, published online 1 June 2020.

The original version of this Article contained an error in Figure 2, where the horizontal axis value ‘1600’ in panel (a) was incorrectly labelled as ‘1200’. This has now been corrected in both the PDF and HTML versions of the Article.

