# Supplementary material for: Ultrahigh high-strain-rate superplasticity in a nanostructured high-entropy alloy
Source: Nat Commun. 2020 Jun 1;11:2736. doi: 10.1038/s41467-020-16601-1 (PMC7264233; doi:10.1038/s41467-020-16601-1)

Supplementary Information

Ultra-high high-strain rate superplasticity in a nanostructured high-entropy alloy

Nguyen[1] & Asghari-Rad[1] et al.

Supplementary Figure 1 | Scanning transmission electron microscopy (STEM) image from 5 turns HPT processed $Al_9(CoCrFeMnNi)_{91}$ (at%) HEA showing nanocrystalline FCC matrix with average grain size of ~30 nm.

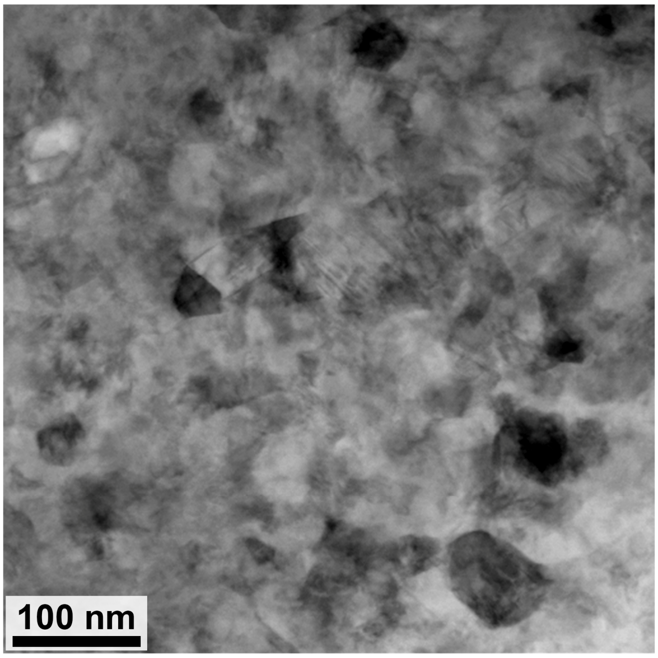

Supplementary Figure 2 | The images of fractured specimens with their testing parameters.

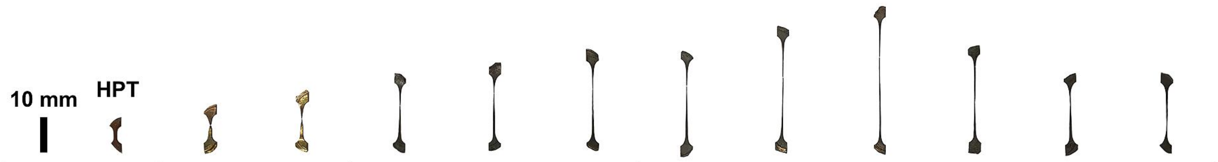

Temperature (K)	873		973			1073					
Strain rate (s^{-1})	$5x10^{-1}$	10^{-2}	$5x10^{-1}$	10^{-1}	10^{-2}	$5x10^{-1}$	10^{-1}	$5x10^{-2}$	10^{-2}	$5x10^{-3}$	10^{-3}
Elongation (%)	250	430	750	1030	1260	1230	1600	2000	1265	780	780

Supplementary Figure 3 | Differential scanning calorimetry (DSC) curve of HPT processed $Al_9(CoCrFeMnNi)_{91}$ (at%) HEA.

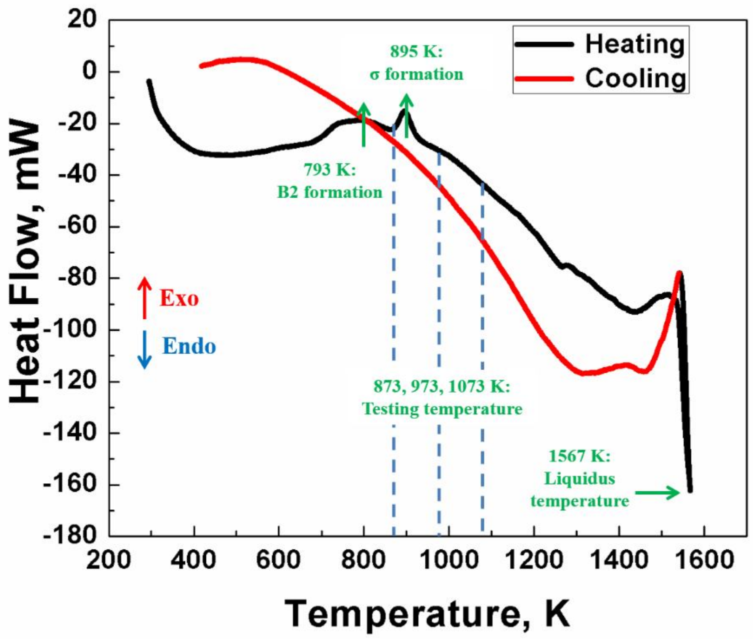

Supplementary Figure 4 | Transmission Electron Microscope - Electron Dispersive X-ray Spectroscopy (TEM-EDS) images and phase maps at the grip, middle, and tip parts on specimen tested at 1073 K and 5×10^{-2} s^{-1}.

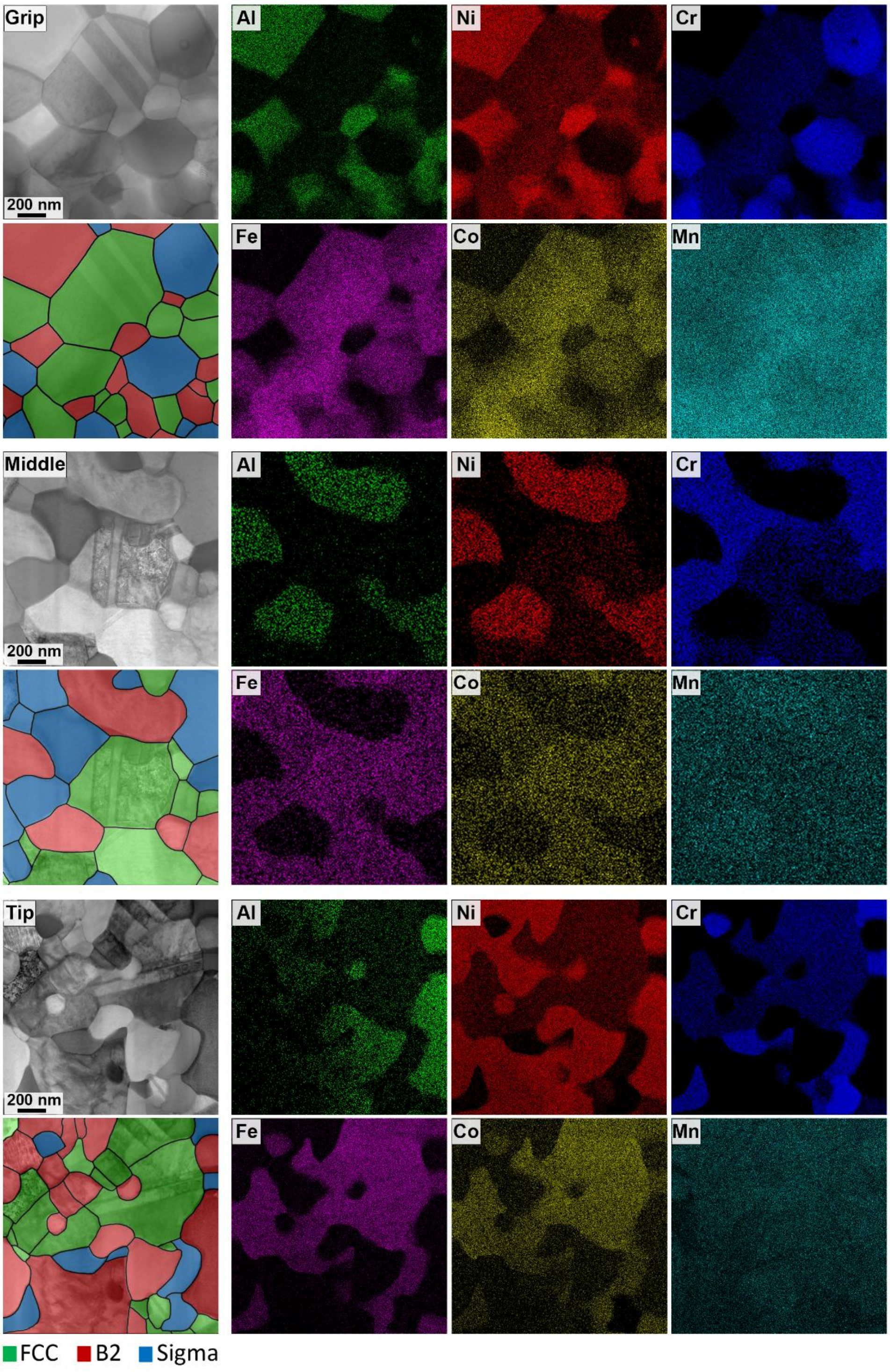

Supplementary Figure 5 | Scanning Transmission Electron Microscopy (STEM) images taken from tip part at two different tilting angles to reveal intragranular dislocations.

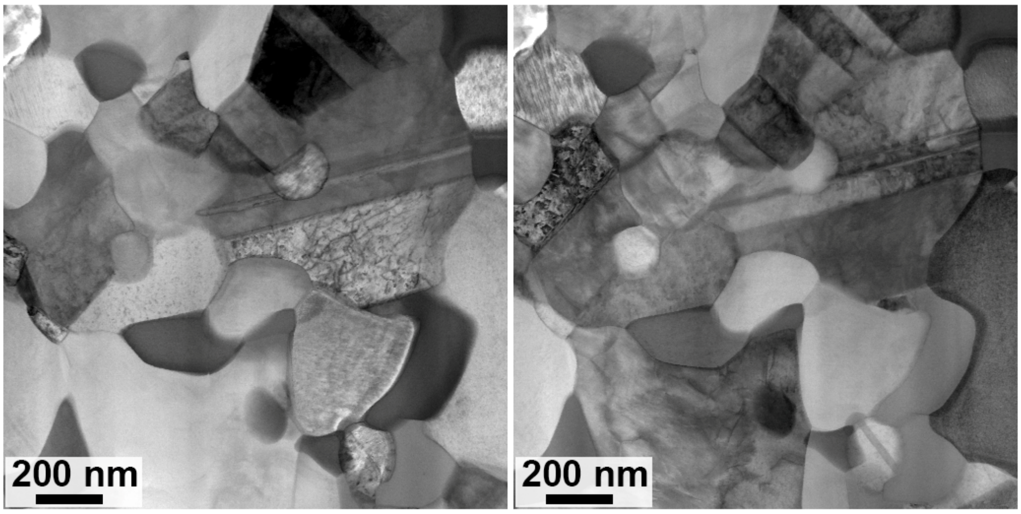

Supplementary Figure 6 | High Resolution (HR)- TEM image and inverse Fast Fourier Transform (FFT) images of $(\bar{2}00)$, $(\bar{1}1\bar{1})$, and $(11\bar{1})$ atomic planes of FCC phase taken from interphase boundary of FCC-B2 phases at (a) the grip part and (b) the tip part of specimen tested at 1073 K and 5×10^{-2} s^{-1}.

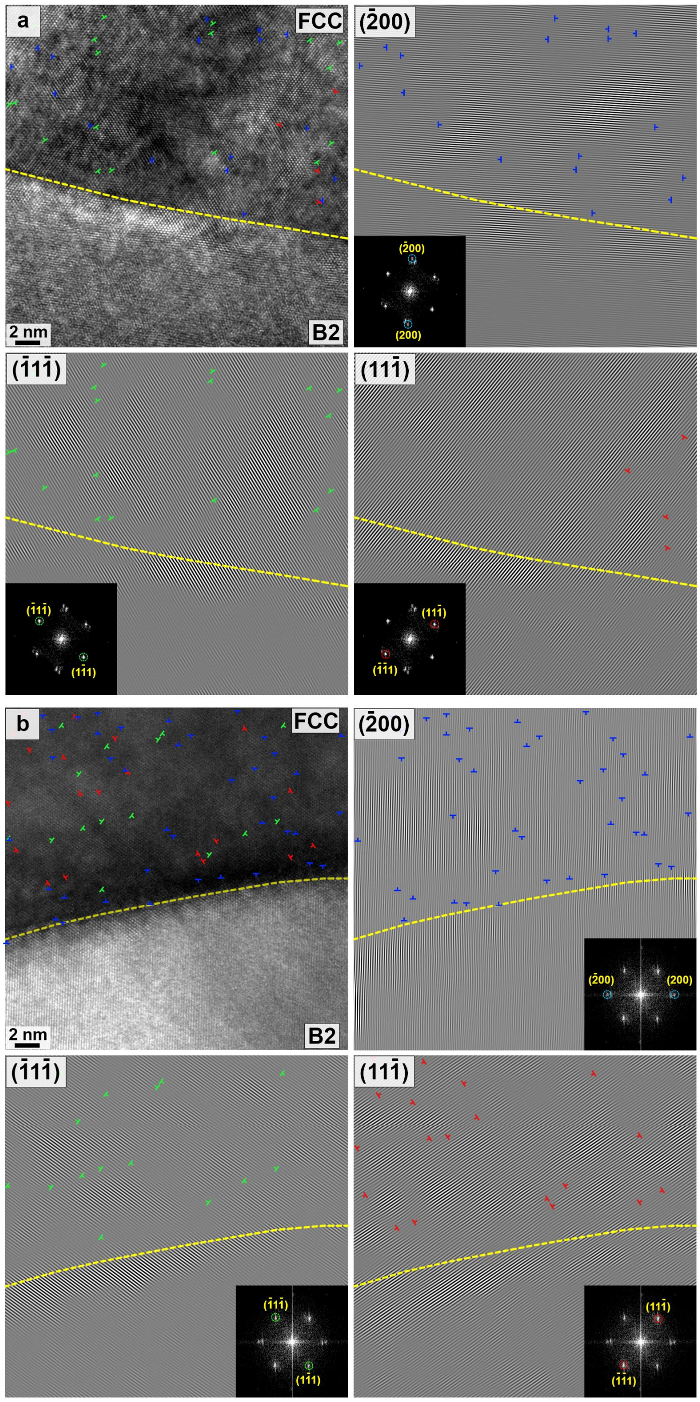

Supplementary Figure 7 | HR-TEM images of (a) FCC-sigma and (b) B2-sigma interfaces with corresponding FFT patterns from marked areas.

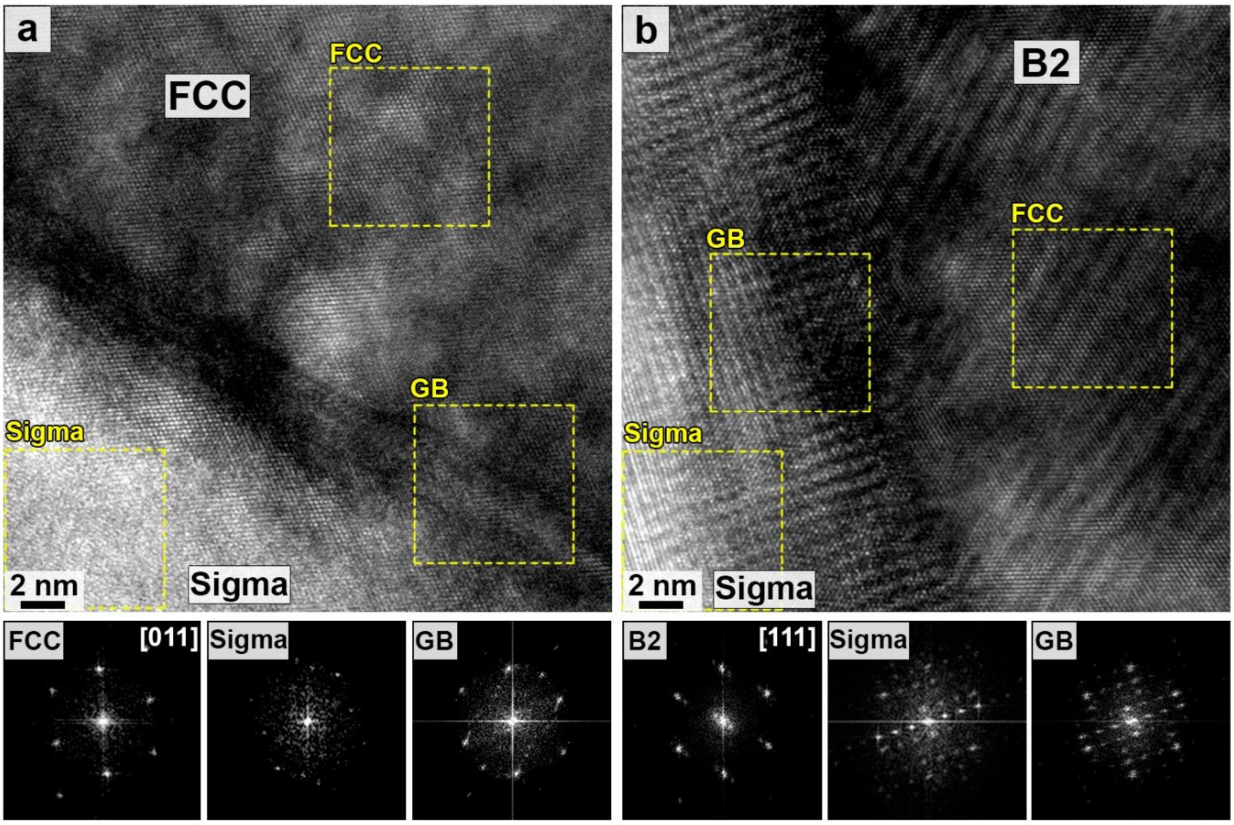

Supplementary Figure 8 | HR-TEM image and inverse FFT images of different atomic planes taken from interface of (a) FCC-sigma and (b) B2-sigma phases at the tip part of specimen tested at 1073 K and 5×10^{-2} s^{-1}.

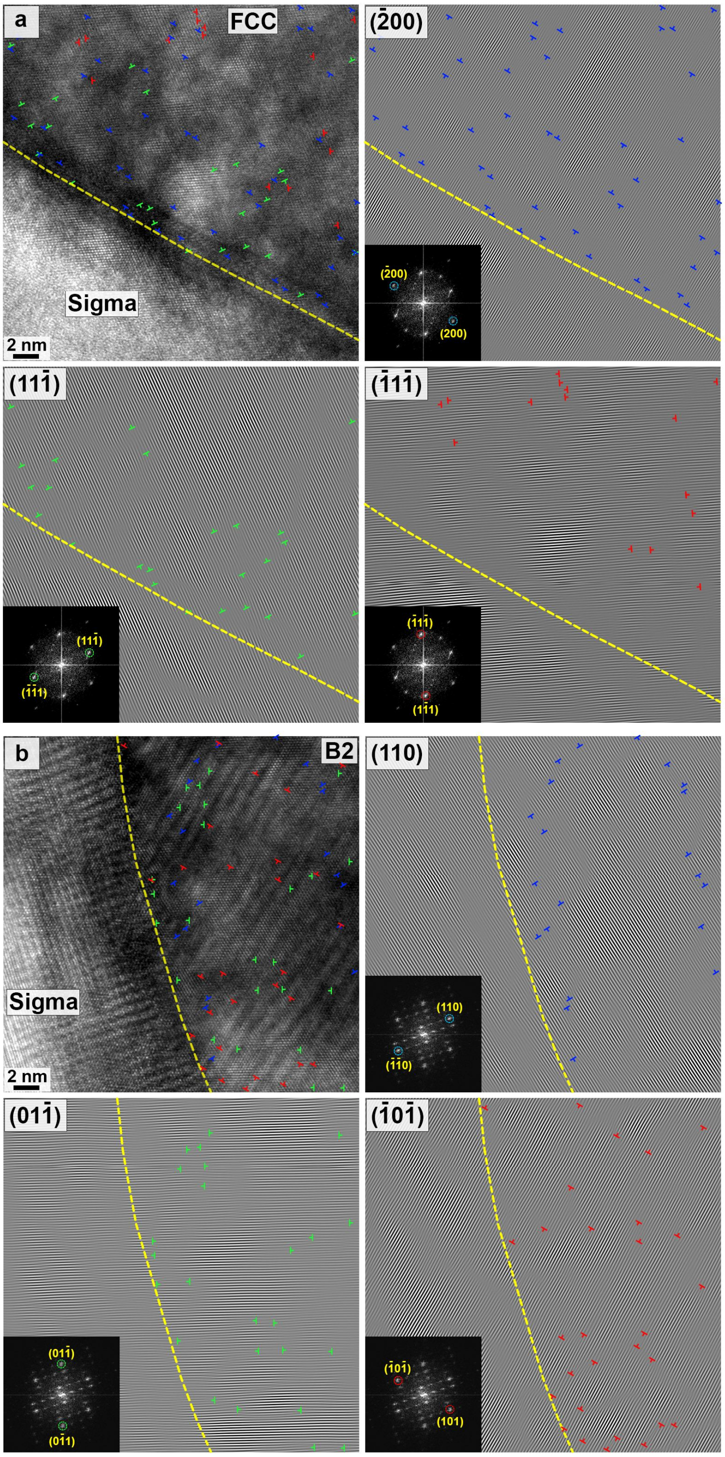

Supplementary Figure 9 | Backscattered Electron (BSE) images and corresponding Scanning Electron Microscope (SEM)-EDS maps from the cavity expansion zone at the tip part on specimen tested at 1073 K and 5×10^{-2} s^{-1}

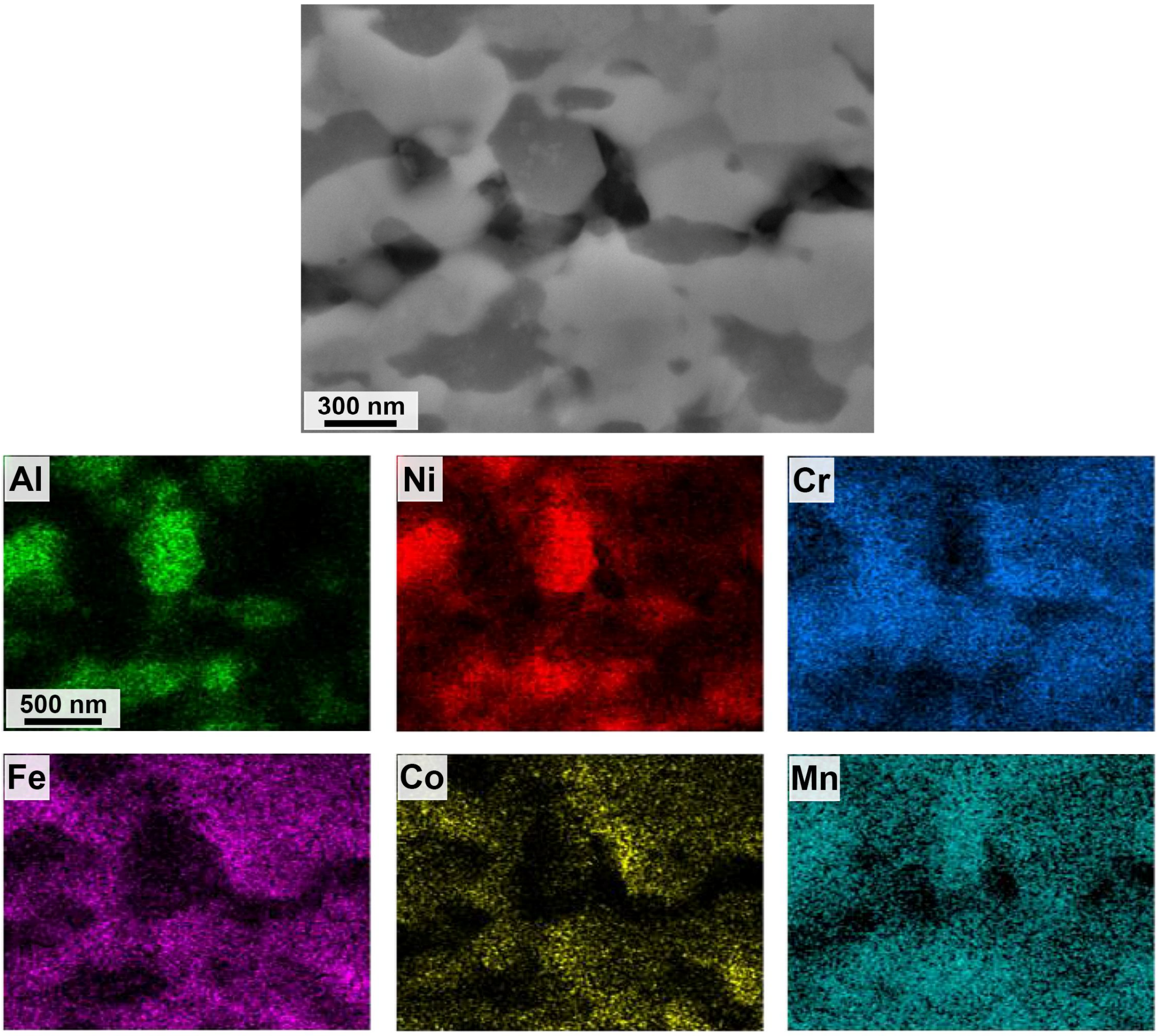

Supplementary Figure 10 | TEM-EDS line-scanning showing evolution of chemical composition across different interface boundary at the tip part on specimen tested at 1073 K and $5{\times}10^{-2}$ s^{-1}. (a) FCC-FCC, (b) FCC-B2, (c) FCC-Sigma, and (d) B2-Sigma interface boundaries.

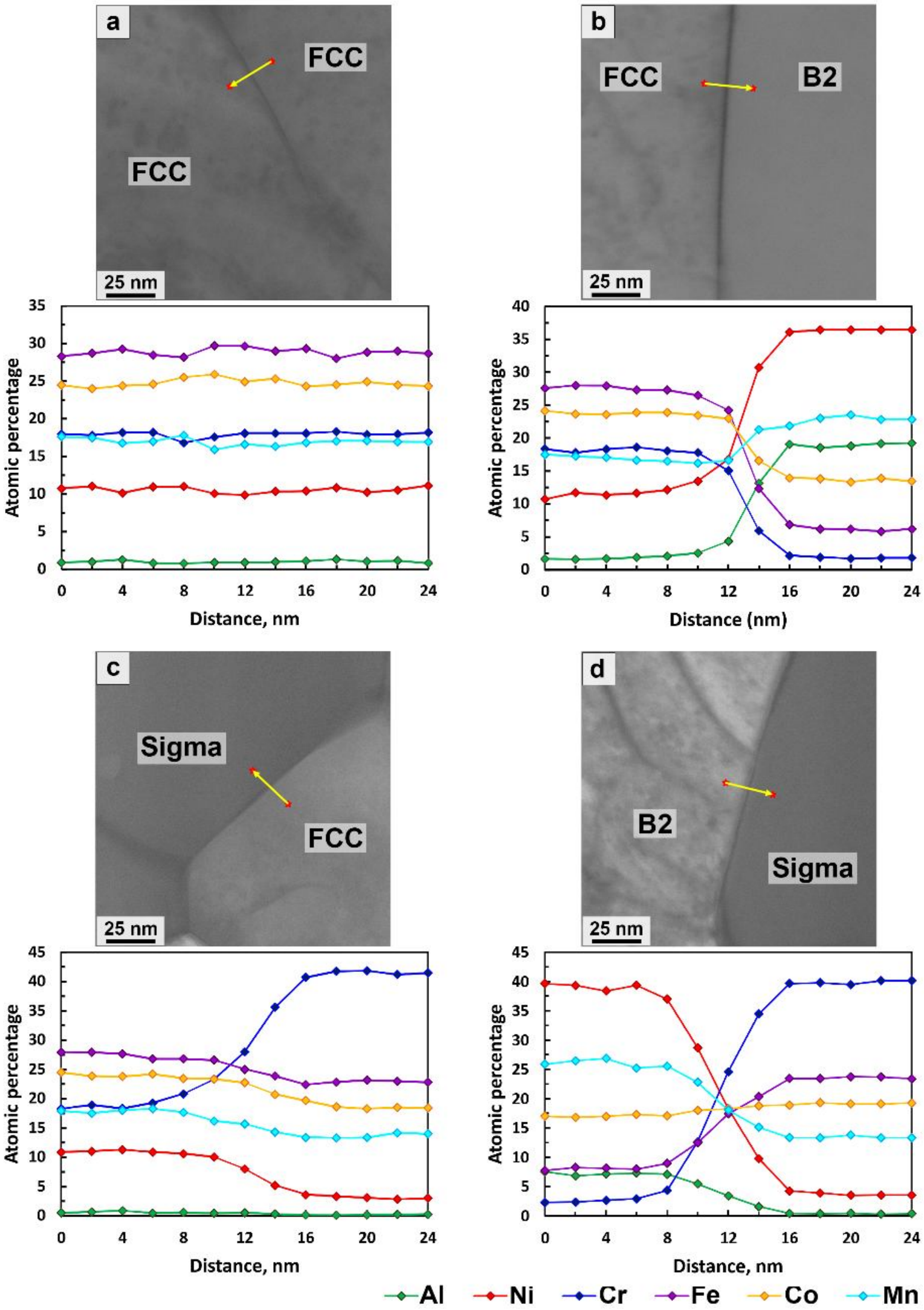

Supplement: Supplementary file 1 — Supplementary Information [file 41467_2020_16601_MOESM1_ESM.pdf]
